# CCL20 expression is elevated in inflammatory bowel disease and attenuated by vitamin D metabolites

**DOI:** 10.1038/s41598-025-05094-x

**Published:** 2025-06-20

**Authors:** Johannes Stallhofer, Felix Reichl, Michael Lauseker, Lisa Waldenmaier, Helga Paula Török, Julia Mayerle, Torsten Olszak, Fabian Schnitzler, Iris Frasheri, Simone Breiteneicher, Stephan Brand, Andreas Stallmach, Julia Diegelmann, Florian Beigel

**Affiliations:** 1 Department of Internal Medicine IV, Jena University Hospital, Am Klinikum 1, 07747 Jena, Germany; 2 Department of Medicine II, University Hospital, LMU Munich, Munich, Germany; 3 Institute for Medical Information Processing, Biometry, and Epidemiology, Faculty of Medicine, LMU Munich, Munich, Germany; 4 Department of Ophthalmology, University Hospital, LMU Munich, Munich, Germany; 5 Department of Conservative Dentistry and Periodontology, University Hospital, LMU Munich, Munich, Germany; 6 Department of Gastroenterology, Kantonsspital St. Gallen, St. Gallen, Switzerland

**Keywords:** C–C motif chemokine ligand 20, CCL20, Vitamin D, Inflammatory bowel disease, Crohn’s disease, Ulcerative colitis, Inflammatory bowel disease, Crohn's disease, Ulcerative colitis

## Abstract

Intestinal epithelial overexpression of the Th17 cell chemoattractant CCL20 is implicated in inflammatory bowel disease and influenced by *NOD2* mutations in Crohn’s disease. Vitamin D metabolites have been shown to ameliorate inflammatory bowel disease. Considering *NOD2* mutations in Crohn’s disease, we investigated whether Vitamin D deficiency (serum 25-hydroxyvitamin D concentration < 20 ng/mL) increases circulating CCL20 levels in inflammatory bowel disease patients and healthy controls and whether active 1,25-dihydroxyvitamin D (calcitriol) downregulates systemic and intestinal CCL20 expression. In a cross-sectional study, serum concentrations of CCL20, 25-hydroxyvitamin D, and calcitriol were measured in 170 *NOD2*-genotyped Crohn’s disease patients, 80 ulcerative colitis patients, and 60 healthy controls. Additionally, the effect of calcitriol on experimentally induced CCL20 expression was examined using human intestinal epithelial HT-29 cells. Multivariable linear regression analyses revealed that both the diagnosis of inflammatory bowel disease and vitamin D deficiency were independently associated with elevated CCL20 levels. Compared to healthy controls, Crohn’s disease patients and ulcerative colitis patients exhibited significantly higher circulating CCL20 levels. Unlike in Crohn’s disease patients, vitamin D deficiency was associated with higher CCL20 levels in healthy controls and ulcerative colitis patients, whereas the calcitriol/25-hydroxyvitamin D activation ratios were negatively correlated with serum CCL20 levels in healthy controls and ulcerative colitis patients with sufficient serum 25-hydroxyvitamin D status. Furthermore, calcitriol markedly inhibited intestinal epithelial induction of CCL20. In Crohn’s disease patients, cholecalciferol supplementation was associated with lower serum CCL20 levels, which were unaffected by *NOD2* mutations. These findings suggest that although vitamin D metabolites may downregulate CCL20 expression in healthy controls and ulcerative colitis patients, this regulatory effect appears to be impaired in Crohn’s disease patients.

**Supplementary Information:**

The online version contains supplementary material available at 10.1038/s41598-025-05094-x.

## Introduction

Crohn’s disease and ulcerative colitis are complex and multifactorial disorders of the intestine, representing the two major types of inflammatory bowel disease (IBD)^1^. The prevalence of IBD is expected to rise, with approximately 1% of the population in industrialized countries projected to be affected by 2030^2^. Although the pathogenesis of IBD is poorly understood, it is assumed that an exaggerated mucosal immune response to the gut microbiota causes chronic relapsing intestinal inflammation in genetically susceptible individuals^1^.

A recent umbrella review of meta-analyses has emphasized the significance of vitamin D (VD) deficiency as one of several environmental factors that increase the risk of developing IBD^3^. Accordingly, a long-term prospective cohort analysis from the Nurses’ Health Study, involving 72,719 women in the United States, has demonstrated that a lifestyle associated with higher predicted plasma levels of 25-hydroxyvitamin D is linked to a reduced incidence of Crohn’s disease. Furthermore, each 100-IU/day increase in total cholecalciferol intake from diet and supplements was associated with a 10% decrease in the risk of developing ulcerative colitis^4^. VD deficient women with predicted 25-hydroxyvitamin D levels below 20 ng/mL had a significantly higher risk of developing Crohn’s disease compared to those with levels above 30 ng/mL^4^. Furthermore, data from studies conducted on VD receptor knockout mice have indicated that only the intestinal epithelial expression of the VD receptor is sufficient to protect mice from experimental colitis^5^.

The intestinal epithelium is the main source of cysteine–cysteine (c–c) motif chemokine ligand 20 (CCL20), also known as macrophage inflammatory protein 3 alpha (MIP-3-alpha) or liver and activation-regulated chemokine (LARC). This chemoattractant is responsible for driving the migration of proinflammatory T helper 17 (Th17) cells to the gut via the CCR6 receptor^6–13^. In patients with active IBD, the expression of intestinal epithelial CCL20 is massively upregulated in colonic biopsies, with an increase of more than 200 folds, induced by various toll-like receptor (TLR) ligands and Th17 cytokines^14–16^. Multiple studies, including ours, have established the significant role of Th17 cytokines and certain single nucleotide polymorphisms encoding these cytokines in the pathogenesis of IBD^17–27^.

Active 1,25-dihydroxyvitamin D (calcitriol) has been shown to inhibit Th17 cell differentiation and function, suppress the expression of the CCL20 receptor CCR6, and ameliorate colitis^28–30^. Furthermore, recent studies have demonstrated that calcitriol can downregulate CCL20 expression in adipocytes, human corneal epithelial cells, and porcine enterocytes^31–33^. Recently, in a randomized, double-blinded, placebo-controlled interventional trial involving healthy human adults, administration of a single high dose of oral cholecalciferol (200.000 IU) remarkably reduced serum CCL20 levels in experimentally induced skin inflammation^34^.

Given the overexpression of the Th17 cell chemoattractant CCL20 in the intestinal epithelium of patients with IBD and the suggested inhibitory effects of calcitriol on CCR6 + Th17 cells and CCL20 expression, we aimed to investigate the systemic serum CCL20 levels in patients with IBD and correlate them with VD status. Additionally, we aimed to explore the impact of calcitriol on systemic serum CCL20 levels and the local CCL20 expression induced by TLR ligands and muramyl dipeptide (MDP) in intestinal epithelial cells^14,35,36^. Since MDP-sensing and subsequent MDP-induced CCL20 expression have been shown to be affected by Crohn’s disease-associated *NOD2* mutations, all patients with Crohn’s disease were genotyped for the three main Crohn’s disease-associated *NOD2* mutations: p.Arg702Trp (rs2066844), p.Gly908Arg (rs2066845), and p.Leu1007fsX1008 (rs2066847). Therefore, any potential influence of Crohn’s disease-associated *NOD2* mutations on CCL20 serum levels was ruled out^35^.

We hypothesized that systemic CCL20 levels might be increased in patients with IBD. We expected a pronounced CCL20 elevation in VD-deficient IBD patients and in VD-deficient healthy individuals, and postulated a downregulation of CCL20 by calcitriol. Based on this hypothesis, a lack of CCL20 suppression could contribute to the development of IBD in VD-deficient individuals. These results would provide a further rationale for investigational studies with VD derivatives as anti-inflammatory agents in IBD patients and for prophylactic cholecalciferol supplementation in their first-degree relatives sharing a genetic predisposition to IBD.

## Results

### Inflammatory bowel disease and vitamin D deficiency are independently associated with elevated CCL20 levels

In the entire study population of 310 individuals, univariable linear regression analyses revealed that IBD diagnosis [β (95% CI) = 0.492 (0.305, 0.678), *p* < 0.0001] and VD deficiency [β (95% CI) = 0.236 (0.076, 0.397), *p* = 0.0040] were associated with elevated systemic CCL20 levels (Table 2). Additionally, age [β (95% CI) = 0.010 (0.004, 0.016), *p* = 0.0009] was found to have a slight modulating effect on CCL20 concentrations, while body mass index [BMI; β (95% CI) = 0.012 (0.006, 0.031), *p* = 0.19] showed some influence on serum CCL20 levels in the univariable prescreening, considering a prescreening *p*-value threshold less than 0.25.

**Fig. 1:**
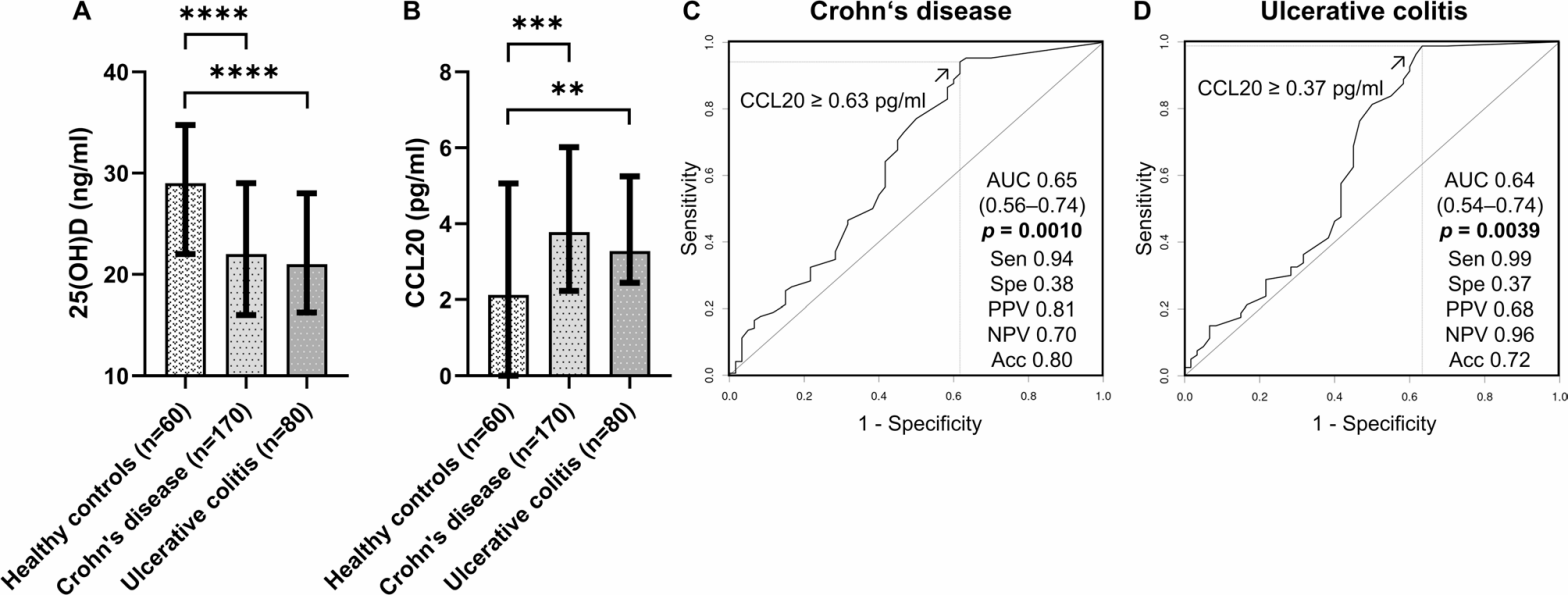
Vitamin D deficiency and systemic upregulation of CCL20 in patients with Crohn’s disease and ulcerative colitis. (**A**) Serum 25-hydroxyvitamin D and (**B**) serum CCL20 levels in healthy controls and patients with Crohn’s disease or ulcerative colitis. Comparisons were performed using the Mann–Whitney U test. **, two-sided *p*-value < 0.01; ***, two-sided *p*-value < 0.001; ****, two-sided *p*-value < 0.0001. Median bar charts with quartile 1, quartile 3, and interquartile range are presented. Raw data for serum 25-hydroxyvitamin D concentrations is shown in Supplementary Table 2. Raw data for serum CCL20 concentrations is shown in Supplementary Table 3. (**C**) Receiver operating characteristic curve showing the diagnostic ability of serum CCL20 to discriminate patients with Crohn’s disease from healthy controls. (**D**) Receiver operating characteristic curve showing the diagnostic ability of serum CCL20 to discriminate patients with ulcerative colitis from healthy controls. Abbreviations: Acc, accuracy; AUC, area under the curve; NPV, negative predictive value; PPV, positive predictive value; Sen, sensitivity; Spe, specificity.

As previously mentioned^37^, VD deficiency was significantly more prevalent in patients with IBD than in healthy controls (HCs; Table 1; Crohn’s disease: 64/170, 38%; ulcerative colitis: 34/80, 43%; IBD: 98/250, 39%; HCs: 5/60, 8%; *p* < 0.0001), and serum 25-hydroxyvitamin D concentrations were significantly lower in patients with Crohn’s disease [median (Q1, Q3): 22.00 (16.00, 29.00) ng/mL, *p* < 0.0001] and ulcerative colitis [median (Q1, Q3): 21.00 (16.25, 28.00) ng/mL, *p* < 0.0001) than in HCs [median (Q1, Q3): 29.00 (22.00, 34.75) ng/mL; Fig. 1A and Supplementary Table 2]. However, an independent and significant association of IBD diagnosis [β (95% CI) = 0.394 (0.197, 0.590), *p* < 0.0001] and VD deficiency [β (95% CI) = 0.168 (0.006, 0.329), *p* = 0.042] with elevated CCL20 levels was confirmed in the multivariable model after adjusting for age [β (95% CI) = 0.0086 (0.002, 0.015), *p* = 0.0070] and BMI [β (95% CI) =  − 0.0035 (− 0.022, 0.015), *p* = 0.71] as additional covariables and including both IBD and VD deficiency in the analysis (Table 2).

**Table 1:** Demographic and clinical characteristics of the study population.

	Patients with Crohn’s disease	Patients with ulcerative colitis	Healthy controls
	(*n* = 170)	(*n* = 80)	(*n* = 60)
	Median (Q1, Q3) or *n* (%)	Median (Q1, Q3) or *n* (%)	Median (Q1, Q3) or *n* (%)
Demographics			
Sex, female	82 (48)	54 (68)	35 (58)
Age, years*	37 (30, 49)	40 (31, 51)	31 (25, 41)
BMI, kg/m^2^	24 (21, 27)	25 (22, 27)	22 (20, 25)
Smokers	55 (35)^a^	7 (10)^b^	3 (5)
Vitamin D deficiency	64 (38)	34 (43)	5 (8)
Vitamin D supplementation, yes	90 (53)	44 (55)	8 (13)
If yes, median daily dose (IE)	1000 (1000, 1000)	1000 (1000, 1000)	2000 (1700, 2250)
Disease duration, years	12 (7, 19)	11 (6, 18)	
Disease activity			
Active	26 (15)	42 (53)	
Remission	144 (85)	38 (48)	
CDAI	60 (28, 100)	–	
CAI	–	4 (2, 5)	
C-reactive protein, mg/dl	0.30 (0.10, 0.80)	0.40 (0.10, 0.80)	
Crohn’s disease location			
Ileum isolated	29 (17)		
Colon isolated	11 (6)		
Ileocolic	90 (53)		
Ileocolic + jejunum	13 (8)		
Ileocolic + upper gastrointestinal tract	27 (16)		
Extent of ulcerative colitis^c^			
Proctitis (E1)		3 (4)	
Left-sided colitis (E2)		35 (44)	
Pancolitis (E3)		38 (48)	
Extraintestinal manifestations	51 (30)	16 (20)	
Fistula	61 (36)	1 (1)	
Stenosis	72 (42)	5 (6)	
Abscess	33 (19)	0 (0)	
Previous surgery	75 (44)	2 (3)	
Current IBD medications			
No IBD medications	15 (9)	–	
Oral steroids	5 (3)	8 (10)	
Immunomodulators^d^	11 (6)	4 (5)	
Anti-TNF therapy	121 (71)	48 (60)	
Anti-TNF therapy + immunomodulators	5 (3)	3 (4)	
Vedolizumab	13 (8)	6 (8)	
5-aminosalicylic acid monotherapy	–	11 (14)	
*NOD2* status			
p.Arg702Trp (HET or HOM; HOM)	28 (16), 2 (1)		
p.Leu1007fsX1008 (HET or HOM; HOM)	40 (24), 9 (5)		
p.Gly908Arg (HET or HOM; HOM)	11 (6), 1 (1)		

*BMI* body mass index, *CAI* clinical activity index (Rachmilewitz), *CDAI* Crohn’s disease activity index, *HET* heterozygous, *HOM* homozygous, *IBD* inflammatory bowel disease, *Q1* first quartile, *Q3* third quartile.

^a^Missing data for 14 patients, ^b^missing data for nine patients, ^c^missing data for four patients, and ^d^azathioprine or methotrexate.

**Table 2:** Vitamin D deficiency and inflammatory bowel disease diagnoses are independently associated with elevated systemic CCL20 levels.

Variables	CCL20 (pg/mL)^a^			
	Entire study population (*n* = 310)			
	Univariable		Multivariable	
	β (95% CI)	*p*	β (95% CI)	*p*
Sex, female	− 0.042 (− 0.193, 0.108)	0.58	0.74	
Age, years	**0.010 (0.004, 0.016)**	**0.00092**	**0.0086 (0.002, 0.015)**	**0.0070**
BMI, kg/m^2^	0.012 (− 0.006, 0.031)	0.19	− 0.0035 (− 0.022, 0.015)	0.71
Smoking	0.012 (− 0.179, 0.203)	0.90	.000013 (0.000058)	
Vitamin D supplementation	0.085 (− 0.069, 0.238)	0.28		
Vitamin D deficiency	**0.236 (0.076, 0.397)**	**0.0040**	**0.168 (0.006, 0.329)**	**0.042**
Inflammatory bowel disease	**0.492 (0.305, 0.678)**	** < 0.0001**	**0.394 (0.197, 0.590)**	**< 0.0001**

Serum CCL20 served as a dependent variable in the entire study population (*n* = 310) in univariable prescreening (threshold *p*-value of less than 0.25) and multivariable linear regression analysis.

*β* unstandardized regression coefficient, *BMI* body mass index, *CI* confidence interval, *p p*-value.

^a^Serum CCL20 levels were log-transformed to achieve a normal distribution of residuals for significance testing.

Significant values are in bold.

### CCL20 is systemically upregulated in Crohn’s disease and ulcerative colitis

Compared to HCs [median (Q1, Q3): 2.12 (0, 5.06) pg/mL], serum CCL20 levels were found to be elevated in patients with Crohn’s disease [median (Q1, Q3): 3.78 (2.23, 5.97) pg/mL, *p* = 0.00041] and ulcerative colitis [median (Q1, Q3): 3.28 (2.45, 5.25) pg/mL, *p* = 0.0036; Fig. 1B and Supplementary Table 3]. At a cutoff value of 0.63 pg/mL or higher, serum CCL20 levels demonstrated optimal diagnostic performance in distinguishing patients with Crohn’s disease from HCs [AUC (95% CI): 0.65 (0.56, 0.74), *p* = 0.0010; sensitivity: 0.94 (0.89, 0.97), specificity: 0.38 (0.26, 0.52), positive predictive value (PPV): 0.81 (0.75, 0.86), negative predictive value (NPV): 0.70 (0.51, 0.84), accuracy: 0.80 (0.74, 0.85); Fig. 1C]. Similarly, to discriminate patients with ulcerative colitis from HCs, an optimal cutoff value of 0.37 pg/mL or higher was determined [AUC (95% CI): 0.64 (0.54, 0.74), *p* = 0.0039; sensitivity: 0.99 (0.93, 1.00), specificity: 0.37 (0.25, 0.50), PPV: 0.68 (0.58, 0.76), NPV: 0.96 (0.78, 1.00), accuracy: 0.72 (0.64, 0.79); Fig. 1D].

### CCL20 is not a disease activity marker for Crohn’s disease and ulcerative colitis

Serum CCL20 levels were found to be comparable between patients with active Crohn’s disease [median (Q1, Q3): 4.08 (2.86, 6.58) pg/mL] and Crohn’s disease in remission [median (Q1, Q3): 3.78 (2.23, 5.69) pg/mL, *p* = 0.23] as well as between patients with active ulcerative colitis [median (Q1, Q3): 3.28 (2.23, 5.54) pg/mL] and ulcerative colitis in remission [median (Q1, Q3): 3.28 (2.50, 5.21) pg/mL, *p* = 0.87]. Serum CCL20 levels did not demonstrate diagnostic ability to distinguish active disease from disease in remission for Crohn’s disease at a cutoff value of 5.64 pg/mL or higher [AUC (95% CI): 0.57 (0.45, 0.70), *p* = 0.23; sensitivity: 0.42 (0.23, 0.63), specificity: 0.74 (0.66, 0.81)] and for ulcerative colitis at a cutoff value of 2.86 pg/mL or higher [AUC (95% CI): 0.51 (0.38, 0.64), *p* = 0.16; sensitivity: 0.62 (0.46, 0.76), specificity: 0.47 (0.31, 0.64)]. Accordingly, linear regression modeling showed no clear association between clinical disease activity and CCL20 levels in patients with Crohn’s disease [univariable: β (95% CI) = 0.242 (− 0.016, 0.500), *p* = 0.066; multivariable: β (95% CI) = 0.210 (− 0.051, 0.471), *p* = 0.11] or ulcerative colitis [univariable: β (95% CI) = 0.083 (− 0.167, 0.333), *p* = 0.51; Table 3]. Likewise, there was no association between CRP levels and CCL20 levels in patients with Crohn’s disease [univariable: β (95% CI) = 0.044 (− 0.023, 0.111), *p* = 0.19; multivariable: β (95% CI) = 0.027 (− 0.040, 0.095), *p* = 0.43] and ulcerative colitis [univariable: β (95% CI) = 0.016 (− 0.121, 0.152), *p* = 0.82; Table 3].

**Table 3:** Vitamin D deficiency is linked to higher systemic CCL20 levels in patients with ulcerative colitis and healthy controls, but not in patients with Crohn`s disease, whereas vitamin D supplementation tends to lower CCL20 levels in patients with Crohn’s disease.

Variables	CCL20 (pg/mL)^a^											
	Patients with Crohn’s disease (*n* = 170)				Patients with ulcerative colitis (*n* = 80)				Healthy controls (*n* = 60)			
	Univariable		Multivariable		Univariable		Multivariable		Univariable		Multivariable	
	β (95% CI)	*p*	β (95% CI)	*p*	β (95% CI)	*p*	β (95% CI)	*p*	β (95% CI)	*p*	β (95% CI)	*p*
Sex, female	− 0.016 (− 0.203, 0.172)	0.87			− 0.109 (− 0.376, 0.157)	0.42			0.018 (− 0.441, 0.477)	0.94		
Age, years	0.0050 (− 0.003, 0.013)	0.20	0.0051 (− 0.003, 0.013)	0.20	0.0090 (− 0.001, 0.018)	0.065	0.0060 (− 0.004, 0.016)	0.23	0.013 (− 0.006, 0.031)	0.18	0.0095 (− 0.009, 0.028)	0.30
BMI, kg/m^2^	0.0060 (− 0.014, 0.026)	0.57			0.018 (− 0.016, 0.053)	0.29			− 0.029 (− 0.100, 0.041)	0.41		
Smoking	− 0.036 (− 0.240, 0.169)	0.73			− 0.296 (− 0.728, 0.137)	0.18	− 0.306 (− 0.731, 0.119)	0.16	− 0.474 (− 1.505, 0.557)	0.36		
Vitamin D supplementation	− **0.188 (**− **0.374, **− **0.003)**	**0.047**	− *0.175 (*− *0.360, 0.010)*	*0.063*	0.065 (− 0.187, 0.316)	0.61			0.523 (− 0.128, 1.174)	0.11	0.441 (− 0.198, 1.080)	0.17
Vitamin D deficiency	0.016 (− 0.178, 0.209)	0.87			0.217 (− 0.026, 0.471)	*0.079*	**0.294 (0.037, 0.550)**	**0.025**	**0.861 (0.074, 1.648)**	**0.033**	**0.796 (0.014, 1.578)**	**0.046**
CRP (mg/dL)	0.044 (− 0.023, 0.111)	0.19	0.027 (− 0.040, 0.095)	0.43	0.016 (− 0.121, 0.152)	0.82						
Active disease	0.242 (− 0.016, 0.500)	0.066	0.210 (− 0.051, 0.471)	0.11	0.083 (− 0.167, 0.333)	0.51						
*NOD2* p.Arg702Trp	− 0.130 (− 0.382, 0.123)	0.31										
*NOD2* p.Gly908Arg	− 0.157 (− 0.536, 0.222)	0.42										
*NOD2* p.Leu1007fsX1008	− 0.030 (− 0.251, 0.191)	0.79										

Serum CCL20 serves as a dependent variable in univariable prescreening (threshold *p*-value of less than 0.25) and multivariable linear regression analyses.

*β* unstandardized regression coefficient, *BMI* body mass index, *CI* confidence interval, *CRP* C-reactive protein, *p p*-value.

^a^Serum CCL20 levels were log-transformed to achieve a normal distribution of residuals for significance testing.

Significant values are in bold, and borderline significant values are in italics.

### Vitamin D deficiency is associated with elevated systemic CCL20 levels in healthy controls and patients with ulcerative colitis, but not in patients with Crohn’s disease, albeit vitamin D supplementation tends to lower CCL20 levels in patients with Crohn’s disease

In univariable linear regression modeling, VD deficiency showed an association with elevated serum CCL20 levels in HCs [β (95% CI) = 0.861 (0.074, 1.648), *p* = 0.033]. This association remained significant in the multivariable model [β (95% CI) = 0.796 (0.014, 1.578), *p* = 0.046], even after adjusting for potential confounders such as age [β (95% CI) = 0.0095 (− 0.009, 0.028), *p* = 0.30] and cholecalciferol supplementation [β (95% CI) = 0.441 (− 0.198, 1.080), *p* = 0.17; Table 3]. In the multivariable linear regression analysis, which included age [β (95% CI) = 0.0060 (− 0.004, 0.016), *p* = 0.23] and smoking status [β (95% CI) =  − 0.306 (− 0.731, 0.119), *p* = 0.16] as covariables, VD deficiency was also found to be associated with increased systemic CCL20 levels in patients with ulcerative colitis [β (95% CI) = 0.294 (0.037, 0.550), *p* = 0.025; Table 3]. In contrast, in patients with Crohn’s disease, VD deficiency had no impact on systemic CCL20 levels, as determined in linear regression modeling [univariable: β (95% CI) = 0.016 (− 0.178, 0.209), *p* = 0.87]. However, cholecalciferol supplementation tended to lower CCL20 levels in patients with Crohn’s disease [univariable: β (95% CI) =  − 0.188 (− 0.374, − 0.003), *p* = 0.047; multivariable: β (95% CI) =  − 0.175 (− 0.360, 0.010), *p* = 0.063; Table 3].

### *NOD2* genotype status has no effect on CCL20 levels in Crohn’s disease

The mutant allele carrier status of the three main Crohn’s disease-associated *NOD2* mutations, p.Arg702Trp [univariable: β (95% CI) =  − 0.130 (− 0.382, 0.123), *p* = 0.31], p.Gly908Arg [univariable: β (95% CI) =  − 0.157 (− 0.536, 0.222), *p* = 0.42], and p.Leu1007fsX1008 [univariable: β (95% CI) =  − 0.030 (− 0.251, 0.191), *p* = 0.79], did not have any significant impact on systemic CCL20 levels in patients with Crohn’s disease (Table 3). In addition, carrying the homozygous p.Leu1007fsX1008 *NOD2* frameshift mutation had no effect on systemic CCL20 levels in patients with Crohn’s disease [univariable: β (95% CI) =  − 0.026 (− 0.443, 0.391), *p* = 0.90]. The distribution of Crohn’s disease-associated variants p.Arg702Trp, p.Gly908Arg, and p.Leu1007fsX1008 was almost identical in Crohn’s disease patients with low (≤ median) and high (> median) CCL20 levels (Supplementary Table 1). Furthermore, absolute CCL20 levels were comparable between *NOD2*-mutant and wildtype patients with Crohn’s disease (Supplementary Fig. 1).

### Higher calcitriol/25-hydroxyvitamin D activation ratios are associated with lower systemic CCL20 levels in healthy controls and ulcerative colitis patients with sufficient vitamin D status

In individuals with sufficient VD status [25-hydroxyvitamin D concentrations ≥ 20 ng/mL], a higher calcitriol/25-hydroxyvitamin D activation ratio showed a negative association with systemic CCL20 levels in HCs [β (95% CI) =  − 0.073 (− 0.117, − 0.028), *p* = 0.0020] and patients with ulcerative colitis [β (95% CI) =  − 0.020 (− 0.036, − 0.004), *p* = 0.017] in the univariable models. This association remained significant in the multivariable regression analyses adjusted for age and vitamin D supplementation in HCs [β (95% CI) =  − 0.068 (− 0.113, − 0.024), *p* = 0.0033] and adjusted for age in ulcerative colitis [β (95% CI) =  − 0.017 (− 0.117, − 0.028), *p* = 0.040]. However, this association could not be detected in patients with Crohn’s disease [univariable: β (95% CI) = 0.014 (− 0.011, 0.038), *p* = 0.26; multivariable: β (95% CI) = 0.010 (− 0.015, 0.035), *p* = 0.43; Table 4].

**Table 4:** Vitamin D activation ratios are associated with lower systemic CCL20 levels in patients with ulcerative colitis and healthy controls.

Variables	CCL20 (pg/mL)^a^											
	Individuals with sufficient vitamin D status [25-hydroxyvitamin D ≥ 20 ng/mL] (*n* = 207)											
	Patients with Crohn’s disease (*n* = 106)				Patients with ulcerative colitis (*n* = 46)				Healthy controls (*n* = 55)			
	Univariable		Multivariable^b^		Univariable		Multivariable^c^		Univariable		Multivariable^d^	
	β (95% CI)	*p*	β (95% CI)	*p*	β (95% CI)	*p*	β (95% CI)	*p*	β (95% CI)	*p*	β (95% CI)	*p*
1,25(OH)_2_D	0.0060 (− 0.002, 0.014)	0.14	0.0055 (− 0.003, 0.014)	0.22	*− 0.0070 (− 0.014, 0.000)*	*0.058*	− 0.0060 (− 0.012, 0.001)	0.11	− 0.0070 (− 0.022, 0.007)	0.29	− 0.0050 (− 0.020, 0.009)	0.44
1,25(OH)_2_D/25(OH)D	0.014 (− 0.011, 0.038)	0.26	0.010 (− 0.015, 0.035)	0.43	− **0.020 (**− **0.036, **− **0.004)**	**0.017**	− **0.017 (**− **0.033, **− **0.001)**	**0.040**	− **0.073 (**− **0.117, **− **0.028)**	**0.0021**	− **0.068 (0.022) (**− **0.113, **− **0.024)**	**0.0033**

Serum CCL20 serves as a dependent variable in univariable and multivariable linear regression analyses, while active 1,25-dihydroxyvitamin D and the 1,25-dihydroxyvitamin D/25-hydroxyvitamin D activation ratio serve as independent variables. Multivariable analyses include sex, age, body mass index, smoking status, CRP, disease activity, and vitamin D supplementation as appropriate and after univariable prescreening (threshold *p*-value of less than 0.25).

*β* unstandardized regression coefficient, *BMI* body mass index, *CI* confidence interval, *CRP* C-reactive protein, *p* p-value.

^a^Serum CCL20 levels were log-transformed to achieve a normal distribution of residuals for significance testing.

^b^Covariates with univariable *p*-values less than 0.25: vitamin D supplementation, CRP, and disease activity.

^c^Covariate with a univariable *p*-value less than 0.25: age.

^d^Covariates with univariable *p*-values less than 0.25: age and vitamin D supplementation.

Significant values are in bold, and borderline significant values are in italics.

### Vitamin D status directly correlates with systemic CCL20 levels in healthy controls

**Fig. 2:**
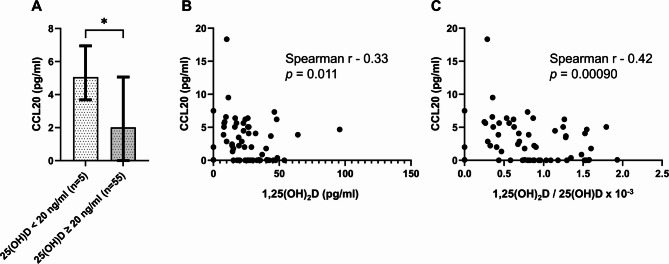
Vitamin D modulates systemic serum CCL20 levels in healthy controls. (**A**) Healthy controls with vitamin D deficiency (25-hydroxyvitamin D concentrations < 20 ng/mL) display higher serum CCL20 levels than healthy controls with sufficient vitamin D status (25-hydroxyvitamin D concentrations ≥ 20 ng/mL). Comparison was performed using the Mann–Whitney U test. *, two-sided *p*-value < 0.05. Median bar charts with quartile 1, quartile 3, and interquartile range are presented. Raw data for CCL20 concentrations is shown in Supplementary Table 4. (**B**) Higher active 1,25-dihydroxyvitamin D (calcitriol) concentrations correlate with lower serum CCL20 levels in healthy controls [Spearman’s rank correlation coefficient (r) =  − 0.33, two-sided *p*-value = 0.011]. (**C**) The 1,25-dihydroxyvitamin D/25-hydroxyvitamin D activation ratios in healthy controls show a high negative correlation with serum CCL20 levels [r =  − 0.42, two-sided *p*-value = 0.00090].

In line with the linear regression analysis results, HCs with VD deficiency [median (Q1, Q3): 5.06 (3.88, 6.39) pg/mL] had significantly higher circulating CCL20 levels than HCs with sufficient 25-hydroxyvitamin D serum status [median (Q1, Q3): 2.02 (0, 4.87) pg/mL, *p* = 0.031; Fig. 2A and Supplementary Table 4]. Accordingly, active calcitriol concentrations were negatively correlated with serum CCL20 levels in HCs (*r* =  − 0.88, *p* = 0.011; Fig. 2B). This negative correlation was even more pronounced for the 1,25(OH)_2_/25-hydroxyvitamin D activation ratio (*r* =  − 0.88, *p* = 0.00090; Fig. 2C). However, in patients with ulcerative colitis, there were no remarkable absolute differences in systemic CCL20 levels between patients with or without VD deficiency, and no direct correlations were found between 1,25(OH)_2_ concentrations or the 1,25(OH)_2_/25-hydroxyvitamin D activation ratios and serum CCL20 levels (data not shown). These findings did not align with the corresponding linear regression modeling results regarding the associations between VD deficiency (Table 3) or 1,25(OH)_2_/25-hydroxyvitamin D activation ratio (Table 4) and serum CCL20 levels in patients with ulcerative colitis. Similarly, in patients with Crohn’s disease, there were no absolute differences in serum CCL20 levels between VD-deficient and sufficient patients, and no direct correlations were observed between 1,25(OH)_2_ concentrations or 1,25(OH)_2_/25-hydroxyvitamin D activation ratios and serum CCL20 levels (data not shown). These findings were consistent with the results of the linear regression models.

### Calcitriol inhibits intestinal epithelial CCL20 expression

**Fig. 3:**
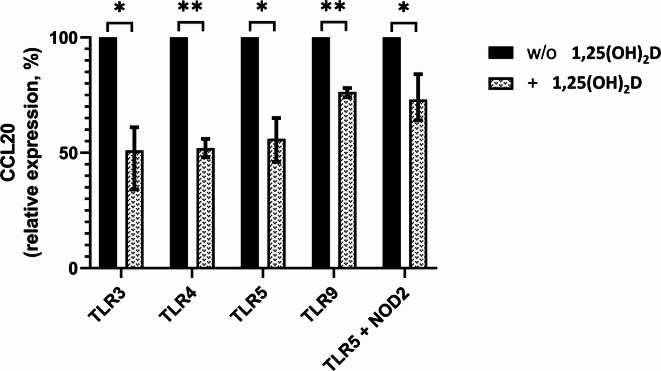
Calcitriol (1,25-dihydroxyvitamin D) inhibits CCL20 expression in intestinal epithelial cells induced by various toll-like receptor ligands and the TLR5/NOD2 antibacterial response loop. Protein expression of CCL20 in human intestinal epithelial HT-29 cells was measured by an enzyme-linked immunosorbent assay in cell culture supernatants after 24 h of stimulation with 10 µg/mL poly(I:C) (a TLR3 ligand), 100 ng/mL lipopolysaccharide (a TLR4 ligand), 1 µg/mL flagellin (a TLR5 ligand), 1 µM ODN2006 (a TLR9 ligand), and 1 µg/mL flagellin in combination with 2 µg/mL muramyl dipeptide (TLR5 + NOD2 ligands), with or without the addition of 100 nM 1,25-dihydroxyvitamin D (calcitriol). Results were obtained from three independent experiments with measurements in triplicate. Bars represent the mean relative CCL20 expression in three independent experiments, while error bars represent the range (minimum–maximum). TLR ligand-induced CCL20 expression without the addition of 1,25-dihydroxyvitamin D was set to 100% for each experiment (black bars). A comparison was performed using the paired Student’s t-test. *, two-sided *p*-value < 0.05; **, two-sided *p*-value < 0.01; TLR, toll-like receptor; w/o, without.

The potent upregulation of epithelial CCL20, observed in active ulcerative colitis and Crohn’s disease, is primarily induced by TLR3 ligation with dsRNA^14^. Furthermore, the TLR4, TLR5, and TLR9 ligands and the antibacterial TLR5/NOD2 response loop have also been shown to induce CCL20 expression in the intestinal epithelium^14,35,38^. Given our serum assay results, which indicated a downregulation of CCL20 by VD metabolites, we investigated whether the addition of calcitriol could inhibit CCL20 expression induced by these TLR ligands and MDP in intestinal epithelial cells. As shown in Fig. 3, in human intestinal epithelial HT-29 cells, the addition of calcitriol markedly reduced CCL20 expression induced by TLR3, TLR4, TLR5, TLR9, and TLR5/MDP to 51% (*p* = 0.029), 52% (*p* = 0.0023), 56% (*p* = 0.015), 76% (*p* = 0.0026), and 73% (*p* = 0.044), respectively. The detailed results of one representative stimulation experiment with absolute CCL20 levels are depicted in Supplementary Fig. 2.

## Discussion

In the present study, we demonstrated for the first time that enhanced local intestinal CCL20 expression in IBD, as previously described^14^, is accompanied by systemically upregulated levels of this crucial gut-derived Th17 cell chemoattractant not only in patients with Crohn’s disease^16^ but also in those with ulcerative colitis. Furthermore, we demonstrated for the first time that VD deficiency, a major environmental risk factor in IBD development^3,4^, is independently associated with increased systemic CCL20 levels. Accordingly, we revealed that active calcitriol can remarkably suppress intestinal epithelial CCL20 expression and that higher calcitriol/25-hydroxyvitamin D activation ratios are correlated with lower systemic serum CCL20 levels.

Interestingly, our study revealed specific associations between VD deficiency and higher CCL20 levels, as well as higher calcitriol/25-hydroxyvitamin D activation ratios and lower serum CCL20 levels, particularly in patients with ulcerative colitis under normal physiological conditions within the IBD population. Since MDP-induced CCL20 expression via the TLR5/NOD2 response loop is known to be influenced by Crohn’s disease-specific *NOD2* mutations, we speculated that *NOD2* mutations might impact systemic CCL20 levels and explain this specificity^35^. However, all patients with Crohn’s disease were genotyped for the three main Crohn’s disease-associated *NOD2* mutations [p.Arg702Trp (rs2066844), p.Gly908Arg (rs2066845), and p.Leu1007fsX1008 (rs2066847)], and any potential influence of *NOD2* genotype status on serum CCL20 levels could be ruled out through linear regression modeling. Additionally, the distribution of genotypes was almost identical between Crohn’s disease patients with low and high serum CCL20 levels, and the absolute levels of CCL20 were comparable among different allele carriers. These findings suggest that the well-known response loop triggered by bacterial flagellin (a TLR5 ligand) and MDP (a NOD2 ligand) via TLR5 and NOD2 ligation is not the sole mechanism for inducing CCL20 expression in intestinal epithelial cells. Skovdahl et al. have shown that the potent upregulation of intestinal epithelial CCL20 expression in active ulcerative colitis and Crohn’s disease is mainly induced by TLR3 ligation with dsRNA^14^. In fact, our stimulation and inhibition assay indicated that CCL20 expression induced by poly(I:C) (a TLR3 ligand), as well as by lipopolysaccharide (a TLR4 ligand) or flagellin (a TLR5 ligand) alone, was even more susceptible to calcitriol-mediated suppression. Therefore, intestinal CCL20 induction via multiple alternative pathways may outweigh genetic defects in the NOD2 pathway in Crohn’s disease. A more intriguing hypothesis to explain the lack of association between higher calcitriol/25-hydroxyvitamin D activation ratios and lower CCL20 levels in patients with Crohn’s disease is the reduced availability of VD receptors in Crohn’s disease, which may hinder the proper downregulation of CCL20 by calcitriol. A marked reduction in VD receptor expression in the intestinal epithelium has been shown in patients with IBD^5,39,40^. This reduction has been associated with increased immune cell infiltration in human IBD biopsies^41^ and has been directly linked to the development of IBD through VD receptor knockout experiments in various established IBD mouse models^5,42^. The deletion of VD receptors in intestinal epithelial cells leads to defective autophagy due to reduced ATG16L1 expression levels and impaired antimicrobial function of Paneth cells^39,42^. Given the role of inadequate ATG16L1-dependent autophagy in driving ileal inflammation in Crohn’s disease, the genetic association of *ATG16L1* polymorphisms with Crohn’s disease, and the unique dysfunction of Paneth cells in patients with Crohn’s disease^27^, it is plausible to suggest that the reduced availability of VD receptors may have a greater relevance to Crohn’s disease-specific inflammatory processes than ulcerative colitis-specific processes^39^. Furthermore, the downregulation of VD receptor expression seems to be slightly more pronounced in Crohn’s disease than in ulcerative colitis^5,40^. Therefore, the reduced availability of VD receptors may better explain the lack of correlation between higher calcitriol/25-hydroxyvitamin D activation ratios and lower systemic CCL20 levels observed in patients with Crohn’s disease. However, the association of cholecalciferol supplementation with lower serum CCL20 levels in patients with Crohn’s disease is in line with this explanation. A randomized controlled trial in healthy adult monozygotic twins revealed that cholecalciferol supplementation at a dose of 2000 IU per day led to a remarkable 60-fold increase in VD receptor gene expression^43^. Although the median cholecalciferol supplementation dose in our Crohn’s disease cohort was lower (1000 IU per day), considering the background of the dramatic induction observed in the aforementioned trial, we speculate that oral cholecalciferol intake may still have a substantial impact on VD receptor expression and VD receptor-dependent signaling, including the CCL20 downregulation. Therefore, we assumed that cholecalciferol supplementation in patients with Crohn’s disease has the potential to restore the disabled VD pathway involved in suppressing CCL20 in Crohn’s disease by reestablishing an appropriate level of VD receptor expression. However, further interventional studies are needed to validate the hypothesis that even lower cholecalciferol supplementation doses can meaningfully impact VD receptor expression and CCL20 regulation.

Our study has several notable strengths. First, it relied on a robust sample size of 310 individuals, including 250 well-characterized adult patients with IBD, with genotype status specifically assessed for the three main disease-associated *NOD2* variants in patients with Crohn’s disease. Second, unlike most studies investigating the role of VD in patients with IBD, our study investigated not only 25-hydroxyvitamin D levels but also the concentrations of the active hormone, calcitriol, to assess the influence of VD on systemic CCL20 levels^44,45^. Serum 25-hydroxyvitamin D, the main circulating metabolite of VD that can be stored in the liver and adipose tissues, serves as a measure of the overall body reservoir of VD, making it the preferred clinical parameter for defining VD deficiency^44^. However, 25-hydroxyvitamin D needs to be converted to its active form, calcitriol, by the enzyme 1-α-hydroxylase. This enzyme is not only present in renal tissues but also in other cells, including intestinal macrophages and epithelial cells, particularly under inflammatory conditions^40,44,46^. The biologically active form, calcitriol, binds specifically to the VD receptor and regulates VD-dependent gene expression^44,46^. To effectively measure VD pathway activity, we used the hormone-to-prohormone calcitriol/25-hydroxyvitamin D activation ratio. This ratio reflects the proportion of systemic VD reserves that are activated for endocrine signaling^47,48^. By normalizing this metabolic ratio to the entire VD reservoir, as expressed by 25-hydroxyvitamin D levels, we were able to reduce confounding factors arising from differential sun exposure or variations in VD intake that may affect 25-hydroxyvitamin D concentrations in the study groups^47,48^. Compared to 25-hydroxyvitamin D or calcitriol alone, the ratio may serve as a superior predictor of VD pathway-dependent outcomes. Notably, higher calcitriol/25-hydroxyvitamin D activation ratios have been associated with higher percentages of regulatory T cells in patients with multiple sclerosis. Furthermore, it has been observed that men with higher calcitriol/25-hydroxyvitamin D activation ratios are more likely to harbor beneficial butyrate-producing bacteria in the gastrointestinal tract, where the VD receptor is highly expressed^47,49^. While several studies have reported a 25-hydroxyvitamin D deficiency in patients with IBD, the 1,25(OH)_2_ levels are actually increased in these patients^37,40^. To rule out the effect of falsely elevated calcitriol levels due to secondary hyperparathyroidism associated with VD deficiency itself, the association of calcitriol concentrations and calcitriol/25-hydroxyvitamin D activation ratios with systemic CCL20 levels was evaluated in subsets of patients with Crohn’s disease, patients with ulcerative colitis, and HCs with sufficient 25-hydroxyvitamin D serum status [25-hydroxyvitamin D ≥ 20 ng/mL] using linear regression models^50^.

Third, we applied rigorous threshold-based univariable and multivariable linear regression modeling approaches to control for potential confounders like IBD diagnosis^37,44^, sex^51^, age^51,52^, BMI^52^, smoking status^53^, cholecalciferol supplementation^43^, disease activity^44,45^, and CRP levels^44,52^ that might influence VD status and CCL20 levels. Furthermore, to minimize confounding resulting from decreased hepatic 25-hydroxylation of cholecalciferol or reduced renal 1-α-hydroxylation of 25-hydroxyvitamin D, patients with liver cirrhosis or kidney failure were excluded from the study^52^. Therefore, the observed associations between VD deficiency and elevated CCL20 levels, as well as higher calcitriol/25-hydroxyvitamin D activation ratios and lower serum CCL20 levels, were not influenced by these potential confounders.

One possible limitation of our study is the lack of endoscopic disease activity assessments or fecal calprotectin measurements. In our study, disease activity was assessed clinically using the CDAI for patients with Crohn’s disease^54^ or the CAI for patients with ulcerative colitis^55^ and biochemically by serum CRP levels^56,57^. However, it should be noted that serum CRP is used to detect endoscopic activity in patients with IBD and has recently been demonstrated to be actually superior to fecal biomarkers in evaluating extensive mucosal inflammation, particularly in ulcerative colitis^56,57^. Accordingly, serum CRP might be the more relevant parameter reflecting potential inflammatory correlations with serum CCL20 in our study. Moreover, a requirement for undergoing endoscopies or bringing stool samples would have greatly reduced patient recruitment. Furthermore, establishing an IBD disease activity biomarker was not the primary focus of our investigation.

Another limitation of our study is the cross-sectional study design. However, the downregulation of CCL20 expression by VD, as suggested by our clinical data, was strongly supported by our cell culture experiments. In these experiments, active calcitriol markedly inhibited CCL20 induction in intestinal epithelial cells by up to 50%. Recent findings from a randomized, double-blind, placebo-controlled interventional trial in healthy human adults demonstrated that oral administration of even a single high dose of 200,000 IU of cholecalciferol led to a marked reduction in serum CCL20 levels^34^.

However, the exact local concentrations of VD metabolites, including calcitriol in intestinal tissue, remain unknown. In our cell culture experiments, we used a relatively high concentration of 100 nM calcitriol, based on pilot studies and previously published research^14,32,58^. Additionally, given its extensive local expression in intestinal inflammation, we assumed that tissue concentrations of calcitriol in the intestine are significantly higher than its systemic serum levels^40^. A key limitation of our study is the absence of precise data on physiological calcitriol concentrations in intestinal tissue. Furthermore, it remains possible that 25-hydroxyvitamin D, at certain concentrations, may exert effects similar to those of calcitriol.

Furthermore, an exact cutoff value for VD deficiency, below which non-musculoskeletal functions such as immunoregulation are critically affected, is not universally accepted. In this study, we defined VD deficiency as a serum 25-hydroxyvitamin D level below 20 ng/mL, a threshold widely used in research and clinical guidelines, including recommendations from the Institute of Medicine^48,50,59^. Supporting this cutoff, a long-term prospective cohort analysis from the Nurses’ Health Study, involving 72,719 women in the United States, demonstrated that women with predicted plasma 25-hydroxyvitamin D levels below 20 ng/mL had a significantly higher risk of developing Crohn’s disease compared to those with levels above 30 ng/mL^4^. Additionally, a study by Ulitsky et al. found that vitamin D deficiency, defined by this 20 ng/mL threshold, was associated with increased disease activity in Crohn’s disease^60^. However, it remains possible that alternative 25-hydroxyvitamin D thresholds may better discriminate disease risk in the context of inflammatory bowel disease (IBD) and correlate more accurately with immune function. HT-29 cells are widely used as an in vitro model for studying intestinal epithelial function, including cytokine and chemokine regulation^61^. While they are derived from human colon adenocarcinoma, they retain key epithelial characteristics, such as responsiveness to inflammatory stimuli^61^. Previous studies have demonstrated their suitability for investigating vitamin D metabolism and its effects on immune signaling^41,61^. However, transformed cell lines may not fully replicate the behavior of primary intestinal epithelial cells in vivo^61^.

As a final limitation, we acknowledge the absence of data on parathyroid hormone levels and dietary calcium intake in our study groups. As a result, the potential influence of a low-calcium diet as a factor that could elevate systemic calcitriol levels and increase the calcitriol/25-hydroxyvitamin D activation ratio may have been underestimated^62^. Conversely, higher serum CCL20 levels could be attributed to a high-calcium diet leading to reduced calcitriol expression.

Given the high prevalence of VD deficiency in patients with IBD and its relevance for IBD pathogenesis and poor disease outcomes, as well as the importance of the CCR6-CCL20 axis in recruiting proinflammatory Th17 cells to the gut in IBD, the downregulation of the Th17 cell chemoattractant CCL20 expression by VD is a relevant finding with therapeutic potential^6,7,9,11,12,14,37,44,45,63^. Although interventional studies investigating cholecalciferol treatment in IBD are scarce, the available evidence is promising^64^. A recent meta-analysis of 18 randomized controlled trials involving 908 patients demonstrated that oral cholecalciferol supplementation can significantly reduce the relapse rate of patients with IBD in remission^64^.

In conclusion, our study demonstrated for the first time that IBD and VD deficiency are independently associated with elevated serum levels of the Th17 cell chemoattractant CCL20. In line with previous colonic biopsy studies in IBD demonstrating increased local intestinal expression^11,14^, we demonstrated a systemic upregulation of CCL20 in both patients with Crohn’s disease^16^ and ulcerative colitis. Like in HCs, we found that VD deficiency was associated with higher serum CCL20 levels in patients with ulcerative colitis. However, in HCs and ulcerative colitis patients with sufficient 25-hydroxyvitamin D serum status, a higher calcitriol/25-hydroxyvitamin D activation ratio correlated with lower systemic CCL20 levels. Interestingly, this correlation could not be found in patients with Crohn’s disease, suggesting a defective VD pathway in Crohn’s disease. However, oral supplementation of cholecalciferol in patients with Crohn’s disease was found to be associated with lower serum CCL20 levels. One can speculate that cholecalciferol supplementation may reactivate VD signaling and induce CCL20 downregulation, potentially by enhancing the particularly low expression of VD receptors in Crohn’s disease. Our findings provide a further rationale for conducting interventional studies to investigate the use of VD derivatives in the management of IBD and may partly explain the increased susceptibility to IBD observed in VD-deficient individuals.

## Methods

### Study population

The cross-sectional study followed the reporting guidelines outlined in the Strengthening the Reporting of Observational Studies in Epidemiology (STROBE) statement (STROBE checklist, Supplementary Checklist 1), as recommended by the Equator Network (https://www.equator-network.org/)^65^. It included a total of 250 adult patients with IBD and 60 unrelated HCs of European Caucasian descent. Within the IBD cohort, 170 patients had Crohn’s disease, and 80 had ulcerative colitis. These patients were consecutively recruited during their visits to the outpatient IBD clinics at the Department of Medicine II, University Hospital, LMU Munich, Germany, between March 2015 and July 2016. The inclusion criteria for the study were being 18 years of age or older and having a confirmed diagnosis of IBD (Crohn’s disease or ulcerative colitis) based on established endoscopic, histological, and clinical criteria according to the guidelines of the German Society for Gastroenterology, Digestive and Metabolic Diseases^66,67^. Exclusion criteria included pregnancy, chronic kidney disease, liver cirrhosis, or clinical evidence of active infection. It is worth noting that another investigation analyzing this IBD cohort has already been published elsewhere^48^. The HCs were individuals without a known history of acute or chronic inflammatory disease, liver disease, kidney disease, or use of anti-inflammatory drugs. They were randomly selected from hospital staff, medical students, and visitors. Clinical disease activity in patients with Crohn’s disease was determined using the Crohn’s disease activity index (CDAI). A CDAI score of 150 or lower indicated disease remission, while a CDAI score above 150 indicated active disease^54^. Disease activity in patients with ulcerative colitis was measured using the clinical activity index (CAI) developed by Rachmilewitz^55^, with a score of 3 or lower indicating disease remission and a score above 3 indicating active disease. Demographic and clinical data (e.g., IBD location, medical treatment, and disease-related complications such as surgery) were obtained from patient charts and interviews at the time of enrollment. The demographic data of HCs and the demographics and clinical characteristics of the Crohn’s disease and ulcerative colitis patient cohorts are summarized in Table 1. All subjects provided written informed consent. The study was approved by the Ethics Committee of the Medical Faculty of LMU Munich as the responsible Institutional Review Board (approval code 343–09) and adhered to the ethical principles of the Helsinki Declaration.

### Sample collection and laboratory analyses

At the time of enrollment, venous blood samples were collected from all study participants into clot activator-coated tubes (S-Monovette®, Sarstedt, Nuembrecht, Germany). Immediately after collection, the tubes were centrifuged at 1000 × *g* for 15 min using a ROTOFIX 32A centrifuge (Hettich, Tuttlingen, Germany), and the obtained serum was stored at − 80 °C in a HFU 586 Basic freezer (Thermo Fisher Scientific, Langenselbold, Germany) until further analysis. The serum CCL20 levels were measured using a commercially available sandwich enzyme-linked immunosorbent assay (Human MIP-3 alpha/CCL20 ELISA Kit; Invitrogen/Thermo Fisher Scientific, Life Technologies Corporation, Carlsbad, United States of America) following the manufacturer’s instructions. Serum 25-hydroxyvitamin D concentrations were measured using the ELECSYS® Vitamin D total II assay kit on a Cobas® e 801 analyzer (Roche Diagnostics, Mannheim, Germany), while serum calcitriol concentrations were measured using the automated chemiluminescence immunoassay kit IDS-iSYS 1,25 VitD^Xp^ on an IDS-iSYS Multi-Discipline Automated System (Immunodiagnostic Systems, Tyne & Wear, United Kingdom) at the Central Facility for Clinical Chemistry, Ulm University Medical Center, Germany. VD deficiency was defined as a serum 25-hydroxyvitamin D concentration below 20 ng/mL, a widely used cutoff value in research and respective guidelines, following the recommendations of the Institute of Medicine^48,50,59^. In patients with IBD, serum C-reactive protein (CRP) levels were measured as part of routine laboratory testing using an automated clinical chemistry analyzer (Beckman Coulter, Krefeld, Germany) at the Institute of Laboratory Medicine, University Hospital, LMU Munich.

### DNA extraction and *NOD2* genotyping in patients with Crohn’s disease

For *NOD2* genotyping, venous blood samples were collected from all patients with Crohn’s disease using ethylenediaminetetraacetic acid as an anticoagulant. Genomic DNA was isolated from peripheral blood leukocytes using the DNA Blood Mini Kit (Qiagen, Hilden, Germany). The *NOD2* exons 4, 8, and 11 were then amplified and sequenced to assess the genotype status for the three main Crohn’s disease-associated variants, i.e., p.Arg702Trp (rs2066844), p.Gly908Arg (rs2066845), and p.Leu1007fsX1008 (rs2066847), as previously described^68^.

### HT-29 cell culture and stimulation experiments

The intestinal epithelial cell line HT-29 (LGC Standards, Wesel, Germany; obtained in passage 128), known to express a functional VD receptor^41^, was cultured in Dulbecco’s modified Eagle medium supplemented with 10% fetal bovine serum (Sigma-Aldrich, Taufkirchen, Germany) and 1% penicillin/streptomycin (Sigma-Aldrich). The cells were incubated in a humidified 5% CO_2_ atmosphere at 37 °C. For stimulation experiments, 5 × 10^5^ HT-29 cells were seeded per well in 24-well plates (Greiner Bio-One, Frickenhausen, Germany) with 0.5 mL of complete medium. The next day, the cells were stimulated in triplicates with various TLR agonists: poly(I:C) (a TLR3 ligand), lipopolysaccharide (a TLR4 ligand), flagellin (a TLR5 ligand), ODN2006 (a TLR9 ligand), or the NOD2 ligand MDP (all obtained from InvivoGen, Toulouse, France). In half of the wells, costimulation with 100 nM calcitriol (Merck, Darmstadt, Germany) was performed, but not in the other half of the wells. The concentrations and incubation times for TLR agonists, MDP, and calcitriol were determined in pilot experiments and were similar to those in previously published studies^14,32,58^. After 24 h, the CCL20 levels were measured in the cell culture supernatants using a commercially available sandwich ELISA kit (Human MIP-3-alpha/CCL20 Quantikine ELISA Kit; R&D Systems/Bio-Techne, Wiesbaden, Germany).

### Statistical analysis

Continuous variables were presented as medians with first and third quartiles (Q1, Q3), while categorical variables were presented as absolute numbers and relative frequencies (*n*, %). To compare groups, nonparametric tests were used, such as the Mann–Whitney *U* test for two groups or the Kruskal–Wallis test for three groups. Results were graphically presented by column bar graphs with medians and interquartile ranges. Spearman’s rank correlation coefficient (*r*) was calculated to correlate between quantitative variables. Frequency distributions of categorical variables on contingency tables were evaluated using Fisher’s exact test. To investigate the association between serum CCL20 levels and VD deficiency and IBD diagnosis, both univariable and multivariable linear regression modeling were performed. Potential confounders such as sex, age, BMI, smoking status, and VD supplementation (“yes” versus “no”) were included in the analysis. In the Crohn’s disease and ulcerative colitis patient cohorts, CRP levels and clinical disease activity (as previously defined) were considered additional covariates. Furthermore, in the Crohn’s disease cohort, the genotype status for the three main Crohn’s disease-associated *NOD2* variants, i.e., p.Arg702Trp (rs2066844), p.Gly908Arg (rs2066845), and p.Leu1007fsX1008 (rs2066847), was included as independent variables (homozygous/heterozygous mutant allele carriers versus homozygous wildtype allele carriers). The selection of independent variables was based on clinical judgment, and those with a *p*-value less than 0.25 in the univariable prescreening were incorporated into the multivariable models. Table 1 provides information on missing data for certain variables in some individuals. Observations with missing values for a particular variable were excluded from the analysis of that variable. Regression analysis results were reported as unstandardized regression coefficient (β) with 95% confidence interval (CI). To ensure that the residuals met the assumption of a normal distribution for significance testing, a log transformation was applied to the serum CCL20 levels when serving as the dependent variable. To assess the ability of serum CCL20 for Crohn’s disease or ulcerative colitis diagnosis, empirical receiver operating characteristics (ROC) curve analyses were performed separately for each cohort. The area under the curve (AUC) was determined, and the optimal diagnostic cutoff value for serum CCL20 levels was identified using Youden’s index. CIs for proportions were calculated using the Clopper-Pearson method. In the HT-29 cell culture experiments, differences in CCL20 expression levels under various stimulation conditions were compared using the paired Student’s *t*-test. All reported *p*-values were two-sided, and a significance level of 0.05 was considered. Due to the exploratory nature of this work, no *p*-value adjustment was made. Linear regression analyses were performed using the Statistical Package for the Social Sciences Statistics (Version 29.0.0.0, IBM, Armonk, New York, United States of America). ROC curve analyses were performed using R software (Version 4.2.2, ROCR, pROC and PropCIs packages). Other graphs were generated using GraphPad Prism 9 (GraphPad Software, San Diego, United States of America), which was also used for all other statistical calculations.

## Electronic supplementary material

Below is the link to the electronic supplementary material.

Supplementary Material 1

Supplementary Material 2

Supplementary Material 3

Supplementary Material 4

Supplementary Material 5

Supplementary Material 6

Supplementary Material 7

## Data Availability

The datasets generated and analyzed during this study are available from the corresponding author upon reasonable request.
