# Supplementary material for: CCL20 expression is elevated in inflammatory bowel disease and attenuated by vitamin D metabolites
Source: Sci Rep. 2025 Jun 20;15:20145. doi: 10.1038/s41598-025-05094-x (PMC12181267; doi:10.1038/s41598-025-05094-x)
Supplement: Supplementary file 2 — Supplementary Material 2 [file 41598_2025_5094_MOESM2_ESM.pdf]

Article: CCL20 Expression Is Elevated in Inflammatory Bowel Disease and Attenuated by Vitamin D Metabolites

Journal: Scientific Reports

Authors: Johannes Stallhofer, Felix Reichl, Michael Lauseker, Lisa Waldenmaier, Helga Paula Török, Julia Mayerle, Torsten Olszak, Fabian Schnitzler, Iris Frasheri, Simone Breiteneicher, Stephan Brand, Andreas Stallmach, Julia Diegelmann, Florian Beigel

Corresponding author: Johannes Stallhofer, Jena University Hospital, Department of Internal Medicine IV, E-mail: johannes.stallhofer@med.uni-jena.de

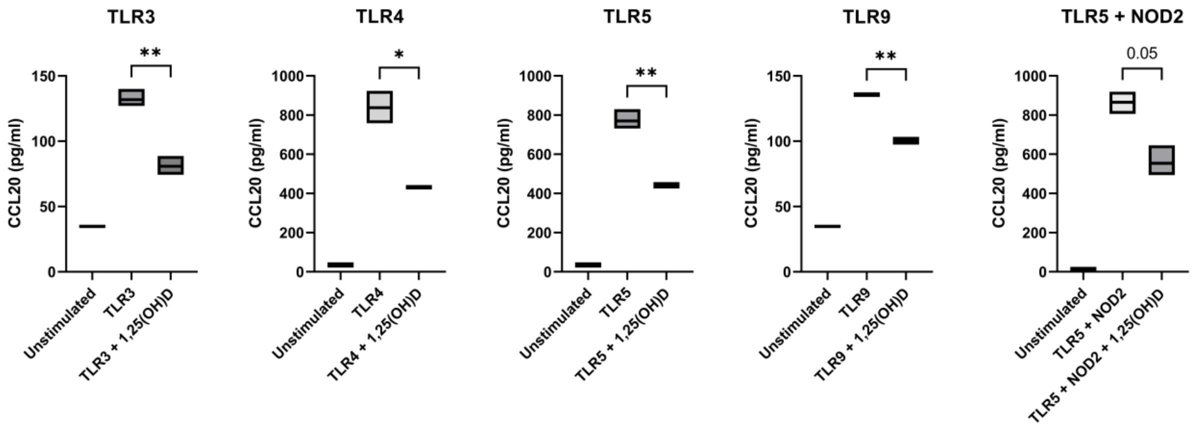

Supplementary Figure 2. Vitamin D inhibits CCL20 expression in intestinal epithelial cells induced by various toll-like receptor ligands

Protein expression of CCL20 in human intestinal epithelial HT-29 cells was measured by an enzyme-linked immunosorbent assay in cell culture supernatants after 24 h of stimulation with 10 µg/mL poly(I:C) (a TLR3 ligand), 100 ng/mL lipopolysaccharide (a TLR4 ligand), 1 µg/mL flagellin (a TLR5 ligand), 1 µM ODN2006 (a TLR9 ligand), and 1 µg/mL flagellin in combination with 2 µg/mL muramyl dipeptide (TLR5 + NOD2 ligands), with or without the addition of 100 nM 1,25-dihydroxyvitamin D. One representative stimulation experiment with absolute CCL20 concentrations is shown in detail. Measurements were done in triplicate. Box plots represent the mean (minimum–maximum) of CCL20 expression. Comparisons were performed using the paired Student's *t*-test. *, two-sided p-value < 0.05; **, two-sided p-value < 0.01; TLR, toll-like receptor
