# Supplementary material for: CCL20 expression is elevated in inflammatory bowel disease and attenuated by vitamin D metabolites
Source: Sci Rep. 2025 Jun 20;15:20145. doi: 10.1038/s41598-025-05094-x (PMC12181267; doi:10.1038/s41598-025-05094-x)
Supplement: Supplementary file 3 — Supplementary Material 3 [file 41598_2025_5094_MOESM3_ESM.pdf]

Article: CCL20 Expression Is Elevated in Inflammatory Bowel Disease and Attenuated by Vitamin D Metabolites

Journal: Scientific Reports

Authors: Johannes Stallhofer, Felix Reichl, Michael Lauseker, Lisa Waldenmaier, Helga Paula Török, Julia Mayerle, Torsten Olszak, Fabian Schnitzler, Iris Frasheri, Simone Breiteneicher, Stephan Brand, Andreas Stallmach, Julia Diegelmann, Florian Beigel

Corresponding author: Johannes Stallhofer, Jena University Hospital, Department of Internal Medicine IV, E-mail: johannes.stallhofer@med.uni-jena.de

Supplementary Table 1. *NOD2* genotype status of Crohn's disease patients with low (≤median) or high (>median) serum CCL20 levels

Using Fisher's exact test, two-sided *p*-values are given for a 3 × 2 contingency table containing homozygous mutant allele carriers and a 2 × 2 contingency table containing the combined subsets of heterozygous and homozygous mutant allele carriers.

Serum CCL20	*NOD2* genotype status								
	p.Arg702Trp (rs2066844)			p.Gly908Arg (rs2066845)			p.Leu1007fsX1008 (rs2066847)		
	CC	CT	TT	GG	GC	CC	*00*	*0*C	CC
Low CCL20 levels (≤median)	68	13	1	77	6	0	64	16	3
High CCL20 levels (>median)	69	12	1	77	4	1	61	16	5
Fisher's exact test, *p*-value	1.00			0.75			0.84		

	CC	CT/TT	GG	GC/CC	00	0C/CC
Low CCL20 levels (≤median)	68	14	77	6	64	19
High CCL20 levels (>median)	69	13	77	5	61	21
Fisher's exact test, *p*-value	1.00		1.00		0.72	
