# Supplementary material for: CCL20 expression is elevated in inflammatory bowel disease and attenuated by vitamin D metabolites
Source: Sci Rep. 2025 Jun 20;15:20145. doi: 10.1038/s41598-025-05094-x (PMC12181267; doi:10.1038/s41598-025-05094-x)
Supplement: Supplementary file 4 — Supplementary Material 4 [file 41598_2025_5094_MOESM4_ESM.pdf]

Article: CCL20 Expression Is Elevated in Inflammatory Bowel Disease and Attenuated by Vitamin D Metabolites

Journal: Scientific Reports

Authors: Johannes Stallhofer, Felix Reichl, Michael Lauseker, Lisa Waldenmaier, Helga Paula Török, Julia Mayerle, Torsten Olszak, Fabian Schnitzler, Iris Frasheri, Simone Breiteneicher, Stephan Brand, Andreas Stallmach, Julia Diegelmann, Florian Beigel

Corresponding author: Johannes Stallhofer, Jena University Hospital, Department of Internal Medicine IV, E-mail: johannes.stallhofer@med.uni-jena.de

Supplementary Table 2. Serum 25-hydroxyvitamin D concentrations in healthy controls and patients with Crohn's disease and ulcerative colitis

Individual serum 25-hydroxyvitamin D concentrations (ng/mL) are presented for 60 healthy controls, 170 patients with Crohn's disease, and 80 patients with ulcerative colitis, as depicted in Figure 1A.

**Healthy controls (n=60)** 25-hydroxyvitamin (ng/mL)	**Patients with Crohn's disease (n=170)** 25-hydroxyvitamin (ng/mL)	**Patients with ulcerative colitis (n=80)** 25-hydroxyvitamin (ng/mL)
79	23	13
62	12	14
52	35	13
58	23	14
38	10	25
31	24	19
49	29	47
19	15	16
39	16	17
35	30	40
19	28	27
39	26	19
32	28	43
22	28	12
34	13	28
31	20	31
33	35	11
31	23	57
26	31	24
44	27	26
22	30	26
27	19	30
34	28	49
58	12	23
50	29	24
35	31	19
31	22	18
34	18	28
22	29	12

22	28	17
25	34	11
32	40	22
23	22	17
22	20	13
22	14	24
34	26	20
21	12	29
36	41	14
25	18	25
28	29	17
28	27	7
23	23	30
35	37	17
27	14	11
25	26	19
30	18	20
31	29	26
25	17	11
25	15	19
31	14	28
21	19	17
20	40	32
32	17	16
15	16	37
26	52	19
15	21	35
18	22	31
21	30	37
21	18	31
20	23	27
	35	35
	16	28
	44	8
	15	21
	12	9
	36	26
	6	29
	35	44
	29	17
	18	12
	19	12
	49	21
	19	20
	10	10
	8	21
	13	23
	27	30

	24	23
	20	20
	21	24
	29	
	21	
	36	
	22	
	9	
	16	
	9	
	15	
	9	
	25	
	12	
	18	
	27	
	20	
	9	
	27	
	24	
	15	
	23	
	13	
	24	
	10	
	10	
	10	
	20	
	17	
	33	
	15	
	12	
	22	
	13	
	27	
	19	
	28	
	22	
	32	
	12	
	42	
	19	
	28	
	16	
	20	
	20	
	13	
	33	

	32	
	20	
	22	
	18	
	19	
	38	
	16	
	23	
	27	
	31	
	14	
	33	
	27	
	54	
	26	
	26	
	41	
	18	
	10	
	50	
	14	
	22	
	22	
	29	
	36	
	18	
	29	
	32	
	23	
	78	
	23	
	22	
	35	
	15	
	16	
	18	
	29	
	14	
	21	
	23	
	41	
	26	
	21	
	31	
	24	
