# Supplementary material for: CCL20 expression is elevated in inflammatory bowel disease and attenuated by vitamin D metabolites
Source: Sci Rep. 2025 Jun 20;15:20145. doi: 10.1038/s41598-025-05094-x (PMC12181267; doi:10.1038/s41598-025-05094-x)
Supplement: Supplementary file 5 — Supplementary Material 5 [file 41598_2025_5094_MOESM5_ESM.pdf]

Article: CCL20 Expression Is Elevated in Inflammatory Bowel Disease and Attenuated by Vitamin D Metabolites

Journal: Scientific Reports

Authors: Johannes Stallhofer, Felix Reichl, Michael Lauseker, Lisa Waldenmaier, Helga Paula Török, Julia Mayerle, Torsten Olszak, Fabian Schnitzler, Iris Frasheri, Simone Breiteneicher, Stephan Brand, Andreas Stallmach, Julia Diegelmann, Florian Beigel

Corresponding author: Johannes Stallhofer, Jena University Hospital, Department of Internal Medicine IV, E-mail: johannes.stallhofer@med.uni-jena.de

Supplementary Table 3. Serum CCL20 concentrations in healthy controls and patients with Crohn's disease and ulcerative colitis

Individual serum CCL20 concentrations (pg/mL) are presented for 60 healthy controls, 170 patients with Crohn's disease, and 80 patients with ulcerative colitis, as depicted in Figure 1B.

**Healthy controls (n=60)** CCL20 (pg/mL)	**Patients with Crohn's disease (n=170)** CCL20 (pg/mL)	**Patients with ulcerative colitis (n=80)** CCL20 (pg/mL)
3.8817	5.0603	6.9557
4.6716	8.792	4.4758
5.6366	7.88	7.327
7.327	9.334	3.071
6.206	8.0633	3.8817
5.6366	10.23	6.0169
6.3944	6.206	5.8272
6.3944	5.2532	6.3944
4.0809	8.792	6.7692
18.346	9.6935	3.276
7.5119	3.276	3.8817
6.206	7.5119	5.6366
9.514	4.6716	6.9557
5.0603	6.0169	14.43
5.8272	8.4285	0.37007
5.6366	7.5119	3.071
0.07685	3.071	4.0809
1.3483	3.8817	2.8643
2.016	5.6366	3.071
2.2318	6.9557	7.88
2.2318	5.8272	2.6556
6.5821	1.7972	2.2318
2.8643	3.6812	2.4449
2.016	6.206	4.866
0	4.6716	0.87849
0	6.7692	1.3483
0	6.3944	2.4449
0	11.822	3.276
0	4.2789	2.8643
0	8.4285	0.87849

0	8.0633	1.7972
0	8.6104	2.6556
0	6.9557	2.4449
0.07685	4.0809	2.016
0	7.5119	3.4793
3.6812	7.6962	0
0.07685	6.0169	2.6556
0.37007	2.8643	3.6812
0.87849	3.6812	3.6812
0	6.5821	3.276
0	4.8664	2.6556
0	6.206	1.7972
0	8.792	3.6812
0	5.8272	9.514
0	6.5821	7.5119
0	0.37007	2.2318
0	9.6935	3.6812
0.07685	4.0809	5.2532
1.7972	2.6556	5.8272
2.8643	4.4758	5.0603
2.4449	3.8817	4.0809
1.7972	3.6812	5.2532
2.016	3.276	5.6366
3.4793	3.8817	7.327
5.0603	4.4758	0.37007
5.0603	5.0603	2.4449
3.8817	0	4.8664
4.0809	2.2318	4.2789
5.0603	3.071	2.2318
4.4758	2.4449	5.2532
	3.071	5.2532
	4.6716	19.355
	3.4793	20.36
	6.0169	3.276
	2.2318	15.972
	2.2318	4.2789
	12.872	2.8643
	4.8664	2.4449
	5.0603	3.276
	1.1167	1.7972
	1.7972	2.016
	2.2318	2.4449
	2.4449	6.206
	3.6812	2.6556
	4.6716	2.8643
	1.5749	1.3483
	2.016	1.5749
	1.7972	1.1167

	5.0603	2.2318
	0.63129	0.87849
	1.3483	
	3.071	
	2.016	
	2.016	
	3.071	
	0	
	5.2532	
	2.2318	
	4.4758	
	0.87849	
	3.071	
	2.6556	
	3.071	
	4.6716	
	4.0809	
	1.5749	
	3.4793	
	6.0169	
	4.4758	
	2.4449	
	2.6556	
	2.8643	
	3.6812	
	4.8664	
	0	
	3.6812	
	2.8643	
	3.071	
	3.6812	
	2.8643	
	3.276	
	7.327	
	0	
	6.0169	
	1.7972	
	2.8643	
	2.016	
	0	
	0.63129	
	7.5119	
	0.63129	
	2.016	
	1.1167	
	3.276	
	0	
	5.2532	

	8.792	
	4.6716	
	5.4453	
	5.4453	
	5.6366	
	6.7692	
	6.0169	
	6.3944	
	4.6716	
	4.4758	
	4.4758	
	12.173	
	3.4793	
	5.2532	
	6.0169	
	6.206	
	0.37007	
	4.4758	
	5.6366	
	5.4453	
	0.63129	
	3.4793	
	2.016	
	2.2318	
	2.8643	
	2.4449	
	3.071	
	4.2789	
	2.6556	
	4.8664	
	0	
	0.87849	
	49.677	
	1.5749	
	1.5749	
	0	
	2.2318	
	1.3483	
	1.5749	
	2.6556	
	0.63129	
	0.87849	
	1.575	
	0.878	
