# Supplementary material for: CCL20 expression is elevated in inflammatory bowel disease and attenuated by vitamin D metabolites
Source: Sci Rep. 2025 Jun 20;15:20145. doi: 10.1038/s41598-025-05094-x (PMC12181267; doi:10.1038/s41598-025-05094-x)
Supplement: Supplementary file 6 — Supplementary Material 6 [file 41598_2025_5094_MOESM6_ESM.pdf]

Article: CCL20 Expression Is Elevated in Inflammatory Bowel Disease and Attenuated by Vitamin D Metabolites

Journal: Scientific Reports

Authors: Johannes Stallhofer, Felix Reichl, Michael Lauseker, Lisa Waldenmaier, Helga Paula Török, Julia Mayerle, Torsten Olszak, Fabian Schnitzler, Iris Frasheri, Simone Breiteneicher, Stephan Brand, Andreas Stallmach, Julia Diegelmann, Florian Beigel

Corresponding author: Johannes Stallhofer, Jena University Hospital, Department of Internal Medicine IV, E-mail: johannes.stallhofer@med.uni-jena.de

Supplementary Table 4. Serum CCL20 concentrations in healthy controls with vitamin D deficiency or sufficient vitamin D status

Individual serum CCL20 concentrations (pg/mL) are presented for 5 healthy controls with vitamin D deficiency (25-hydroxyvitamin D concentrations < 20 ng/mL) and 55 healthy controls with sufficient vitamin D status (25-hydroxyvitamin D concentrations ≥ 20 ng/mL), as depicted in Figure 2A.

**Healthy controls with vitamin D deficiency (25-hydroxyvitamin D < 20 ng/mL) (n=5)** CCL20 (pg/mL)	**Healthy controls with sufficient vitamin D status (25-hydroxyvitamin D ≥ 20 ng/mL) (n=55)** CCL20 (pg/mL)
3.4793	1.7972
5.0603	4.4758
3.8817	0.07685
6.3944	2.4449
7.5119	4.0809
	5.0603
	5.0603
	2.2318
	0
	0
	0.07685
	0
	0
	0
	0
	0.87849
	0
	0.07685
	1.7972
	2.016
	5.0603
	6.5821
	0
	0
	0
	0
	5.6366

	5.6366
	1.3483
	0
	0
	2.8643
	9.514
	0
	2.016
	0.07685
	5.8272
	2.8643
	0
	3.6812
	18.346
	0
	0
	0.37007
	6.206
	4.0809
	6.206
	2.2318
	6.3944
	0
	5.6366
	7.327
	2.016
	4.6716
	3.8817
