# Supplementary material for: CCL20 expression is elevated in inflammatory bowel disease and attenuated by vitamin D metabolites
Source: Sci Rep. 2025 Jun 20;15:20145. doi: 10.1038/s41598-025-05094-x (PMC12181267; doi:10.1038/s41598-025-05094-x)
Supplement: Supplementary file 7 — Supplementary Material 7 [file 41598_2025_5094_MOESM7_ESM.pdf]

Article: CCL20 Expression Is Elevated in Inflammatory Bowel Disease and Attenuated by Vitamin D Metabolites

Journal: Scientific Reports

Authors: Johannes Stallhofer, Felix Reichl, Michael Lauseker, Lisa Waldenmaier, Helga Paula Török, Julia Mayerle, Torsten Olszak, Fabian Schnitzler, Iris Frasheri, Simone Breiteneicher, Stephan Brand, Andreas Stallmach, Julia Diegelmann, Florian Beigel

Corresponding author: Johannes Stallhofer, Jena University Hospital, Department of Internal Medicine IV, E-mail: johannes.stallhofer@med.uni-jena.de

Reporting checklist for cross sectional study.

Based on the STROBE cross sectional guidelines*.

* von Elm E, Altman DG, Egger M, Pocock SJ, Gotzsche PC, Vandenbroucke JP. The Strengthening the Reporting of Observational Studies in Epidemiology (STROBE) Statement: guidelines for reporting observational studies.

		Reporting Item	Page Number
**Title and abstract**			
Title	#1a	Indicate the study's design with a commonly used term in the title or the abstract	1-2
Abstract	#1b	Provide in the abstract an informative and balanced summary of what was done and what was found	2
**Introduction**			
Background / rationale	#2	Explain the scientific background and rationale for the investigation being reported	3-4
Objectives	#3	State specific objectives, including any prespecified hypotheses	4

Methods

Study design	#4	Present key elements of study design early in the paper	15-19
Setting	#5	Describe the setting, locations, and relevant dates, including periods of recruitment, exposure, follow-up, and data collection	15-16
Eligibility criteria	#6a	Give the eligibility criteria, and the sources and methods of selection of participants.	15
	#7	Clearly define all outcomes, exposures, predictors, potential confounders, and effect modifiers. Give diagnostic criteria, if applicable	15-18
Data sources / measurement	#8	For each variable of interest give sources of data and details of methods of assessment (measurement). Describe comparability of assessment methods if there is more than one group. Give information separately for exposed and unexposed groups if applicable.	15-19
Bias	#9	Describe any efforts to address potential sources of bias	15-19, 12-13
Study size	#10	Explain how the study size was arrived at	15
Quantitative variables	#11	Explain how quantitative variables were handled in the analyses. If applicable, describe which groupings were chosen, and why	15-19, 12-13
Statistical methods	#12a	Describe all statistical methods, including those used to control for confounding	18-19
Statistical methods	#12b	Describe any methods used to examine subgroups and interactions	15-19, 12-13
Statistical methods	#12c	Explain how missing data were addressed	18, 27
Statistical methods	#12d	If applicable, describe analytical methods taking account of sampling strategy	15-19
Statistical methods	#12e	Describe any sensitivity analyses	18-19, 5-6

Results

Participants	#13a	Report numbers of individuals at each stage of study—eg numbers potentially eligible, examined for eligibility, confirmed eligible, included in the study, completing follow-up, and analysed. Give information separately for for exposed and unexposed groups if applicable.	15, 5-9, 27-30
Participants	#13b	Give reasons for non-participation at each stage	15, 5-9, 27-30, 10-14
Participants	#13c	Consider use of a flow diagram	-
Descriptive data	#14a	Give characteristics of study participants (eg demographic, clinical, social) and information on exposures and potential confounders. Give information separately for exposed and unexposed groups if applicable.	15, 27, 18, 12, 13
Descriptive data	#14b	Indicate number of participants with missing data for each variable of interest	27
Outcome data	#15	Report numbers of outcome events or summary measures. Give information separately for exposed and unexposed groups if applicable.	5-9, 26-30
Main results	#16a	Give unadjusted estimates and, if applicable, confounder-adjusted estimates and their precision (eg, 95% confidence interval). Make clear which confounders were adjusted for and why they were included	5-9, 27-30, 18, 12-13
Main results	#16b	Report category boundaries when continuous variables were categorized	5-9, 26-30
Main results	#16c	If relevant, consider translating estimates of relative risk into absolute risk for a meaningful time period	-
Other analyses	#17	Report other analyses done—e.g., analyses of subgroups and interactions, and sensitivity analyses	5-9, 26-30

Discussion

Key results	#18	Summarise key results with reference to study objectives	10-14

Limitations	#19	Discuss limitations of the study, taking into account sources of potential bias or imprecision. Discuss both direction and magnitude of any potential bias.	13-14
Interpretation	#20	Give a cautious overall interpretation considering objectives, limitations, multiplicity of analyses, results from similar studies, and other relevant evidence.	10-14, 14
Generalisability	#21	Discuss the generalisability (external validity) of the study results	10-14
**Other Information**			
Funding	#22	Give the source of funding and the role of the funders for the present study and, if applicable, for the original study on which the present article is based	25

The STROBE checklist is distributed under the terms of the Creative Commons Attribution License CC-BY. This checklist can be completed online using https://www.goodreports.org/, a tool made by the EQUATOR Network in collaboration with Penelope.ai
